# Discoveries Interview: Professor Christian Weber on the inflammation in cardiovascular diseases

**DOI:** 10.15190/d.2015.02

**Published:** 2015-02-10

**Keywords:** Professor Christian Weber, interview

**Figure 1:**
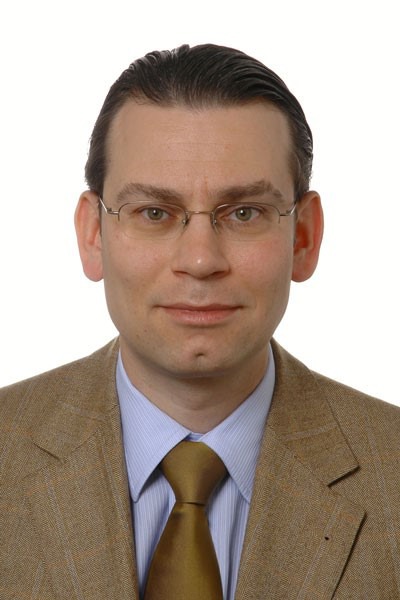
Professor Christian Weber

**Professor Christian Weber **studied medicine at the Ludwig Maximilians – University (LMU) of Munich, Germany, the University Hospital Galway, Ireland and Princess Alexandra Hospital, Australia. After receiving his medical degree in 1994, he joined the Center for Blood Research (T. Springer), Harvard Medical School for a postdoctoral research fellowship. Coming back to Germany, he was awarded as junior research group leader in Vascular Medicine at the Ludwig-Maximilians-University and later was appointed as full-time professor and head of Molecular Cardiology at the University Hospital of the RWTH Aachen. As founding Director of the Institute for Molecular Cardiovascular Research in Aachen, he became a prominent and well-known figure in cardiovascular field, launching well-funded hypothesis and opening novel visions on therapeutic strategies for cardiovascular diseases. In 2010, he was appointed as Chair in Vascular Medicine and Director of the Institute for Cardiovascular Prevention, the Ludwig - Maximilians - University of Munich, as well as Chair of the August-Lenz-Foundation.

During his career, he published more than 400 scientific articles, with a cumulative impact factor of over 3100 and more than 20 000 citations, achieving the h-index of 74. His activity was recognized and awarded by the German Society of Atherosclerosis Research (W.H. Hauss-Prize), the European Society of Cardiology (Outstanding Achievement Award 2008), the American Heart Association (ATVB Special Recognition Award) and the Netherlands Organization for Scientific Research (VICI Award). He is the Chief Editor, Editorial Board Member and/or Referee of many prestigious scientific journals, as well as the president and/or important organizer of several scientific meetings. At last, but not at least, he is an appreciated model and mentor for many ambitioned and motivated young scientists.

## 1. Can you define in simple words what inflammation is, its role in cardiovascular disease and why studying it is so important?

Inflammation is a protective or adaptive response of the immune system against pathogens or tissue damage. The clinical signs of acute inflammation are *dolor, calor, rubor, tumor* and *function laesa*. Basically, these symptoms can be explained by high production of pro-inflammatory mediators, recruitment of leukocyte subsets and increased vascular permeability. The role of inflammation in cardiovascular disease is immense. For instance, atherogenesis and plaque growth are driven by imbalanced lipid metabolism and chronic inflammatory processes involving the innate and adaptive immune system^1^. Therefore, studying inflammation is fundamental to understand the complex pathogenesis of cardiovascular disease and to develop novel biomarker panels together with more effective therapeutics.

## 2. How did our knowledge on inflammation in cardiovascular diseases evolve over the time?

Before, the central role of monocytes, macrophages and innate versus adaptive immunity in cardiovascular disease have been extensively discussed. Recently, new aspects on monocyte and macrophage heterogeneity with differential subset-specific recruitment and hitherto less appreciated cellular players such as neutrophils, dendritic, mast and regulatory T-cells become more evident^2^. The research on platelets, chemokine-receptor pairs and heterophilic chemokine interactions during inflammation is intensively developing as well in the recent past^3^.

## 3. Why is translation into clinic so challenging?

The translation of basic research into clinic is a huge challenge with several pitfalls and very heavy responsibility. Unraveling the molecular mechanisms of inflammation could catalyze the development of innovative cardiovascular therapeutics^4^. However, just a fractional amount of the comprehensive experimental (animal) data shows indeed relevance in clinical setting. The exact risk-benefit prediction is often obscure and associated with adverse side effects. The versatile experimental animal models are similar but largely not identical to men and the development of new therapeutics takes a long time with numerous clinical phases until approval.

## 4. What will the atherosclerosis field look like in 5-10 years?

The atherosclerosis field is so vast that attempting to comprehensively cover all aspects will be futile. It will be rather crucial for the future to identify key determinants that lead to chronic inflammation in atherosclerosis, to dissect the intriguing diversity of lipid-related pro-inflammatory versus homeostatic and atheroprotective pathways, to review and validate genetic variants contributing to cardiovascular disease and to discuss promising clinical applications of selectively and local targeting inflammation depending on disease stage^5^.

## 5. What advice do you have for young scientists?

Science is a cruel mistress, so do not take shortcuts and be prepared for frustration. It will always be “per aspera ad astra”. But on your way, look out for the right mentor. That will help.
